# Galectin Plasmatic Levels Reveal a Cluster Associated with Disease Aggressiveness and Kidney Damage in Multiple Myeloma Patients

**DOI:** 10.3390/ijms252413499

**Published:** 2024-12-17

**Authors:** Lidiane Vasconcelos do Nascimento Carvalho, Reijane Alves Assis, Claudio Montenegro, Michelle Melgarejo da Rosa, Michelly Cristiny Pereira, Maira Galdino da Rocha Pitta, Moacyr Jesus Barreto de Melo Rêgo

**Affiliations:** 1 Research Center for Therapeutic Innovation (NUPIT-SG), Federal University of Pernambuco, Recife 50670-901, PE, Brazil; lidianev.n.carvalho@gmail.com (L.V.d.N.C.); claudio.montenegrojunior@ufpe.br (C.M.); michelle.rosa@ufpe.br (M.M.d.R.); michelly.pereira@ufpe.br (M.C.P.); maira.pitta@ufpe.br (M.G.d.R.P.); 2 Hematology Service of the Hospital de Câncer de Pernambuco, Cabugá/ 50040-000, Recife 50040-000, PE, Brazil; reijaneassis22@gmail.com

**Keywords:** galectin 1, galectin 3, galectin 7, galectin 9, hematology, multiple myeloma

## Abstract

Multiple myeloma (MM) is a malignant disease characterized by the proliferation of plasma cells, primarily in the bone marrow. It accounts for approximately 1% of all cancers and 10% of hematologic malignancies. Clinical manifestations include hypercalcemia, anemia, renal failure, and bone lesions. The pathogenesis of MM involves complex interactions between myeloma cells and their microenvironment. Galectins, a family of β-galactoside-binding proteins, particularly galectin-1, -3, -4, -7, and -9, have been implicated in MM development. This study aimed to assess the plasma levels of these galectins in newly diagnosed MM patients and explore their correlation with clinical parameters. Peripheral blood samples were collected from patients at the Oncohematology Service of the Hospital de Câncer de Pernambuco, and galectin levels were measured using ELISA. Plasma levels of galectins-3, -7, and -9 were significantly higher in MM patients compared to the control group. Three clusters of MM patients were identified based on galectin plasma levels, with cluster 3, characterized by high levels of galectin-1, -4, and -7, being associated with a worse prognosis. A strong positive correlation was found between galectin-1, -4, and -7 levels and markers of kidney function (urea, creatinine, and β2-microglobulin), while negative correlations were observed with hematocrit and hemoglobin. Additionally, galectin-9 showed high accuracy in distinguishing MM patients from healthy controls (AUC = 0.931). Elevated galectin levels were indicative of disease aggressiveness and renal impairment in MM patients. Overall, our findings suggest that galectins-1, -4, -7, and -9 could serve as potential biomarkers for MM progression and severity, warranting further investigation into their utility in MM diagnosis and treatment.

## 1. Introduction

Multiple myeloma (MM) is a clonal hematological malignancy characterized by the uncontrolled proliferation and accumulation of abnormal plasma cells (PC) in the bone marrow (BM). It is the second most common hematological cancer in adults, accounting for 10–15% of cases, following lymphomas. In 2023, an estimated 35,730 Americans were diagnosed with MM, with around 12,590 deaths attributed to the disease [1]. In Brazil, epidemiological data are sparse and largely concentrated in southeastern centers, limiting understanding of regional variation across the country.

Despite advancements in treatment, MM remains incurable, with a median survival of 5–7 years. Prognostic factors include patient characteristics, tumor stage, biological markers, and treatment response [2]. Rising incidence and mortality rates reflect the absence of early diagnostic tools, underscoring the need to explore new biomarkers such as extracellular matrix proteins, angiogenic factors, telomeres, telomerase, and immune markers [3].

Galectins, a family of β-galactoside-binding lectins, have emerged as key players in cancer biology, including MM. These proteins play crucial roles in diverse biological processes, such as tissue repair, adipogenesis, regulation of immune homeostasis, angiogenesis and pathogen recognition. Dysregulation of galectins and their ligands has been associated with several pathological conditions, including cancer, inflammation, infections, fibrosis and metabolic disorders. Through specific interactions with glycans or other proteins, galectins influence the initiation, maintenance and resolution of numerous processes to regulate lymphopoiesis and modulate interactions between hematopoietic and stromal cells [4]. In MM, galectins contribute to tumor growth, metastasis, immune evasion and facilitate interactions between hematopoietic and stromal cells in the bone marrow. These roles make them important targets for therapeutic exploration or manipulation in the context of MM progression and pathogenicity. Elevated galectin-1 (LGALS1) expression in MM CD138+ cells has been associated with poorer patient outcomes, while galectin-3 exhibits variable expression across different lineages and in primary MM cells and has been implicated in angiogenesis and metastasis. Galectin-9 induces apoptosis in T cells, whereas galectin-7 promotes both apoptosis and angiogenesis in different contexts [5,6,7,8]. Several studies have investigated galectins’ roles in the bone marrow microenvironment, particularly in MM cell survival and osteoclastogenesis [9]. Translating these preclinical insights into clinical practice is critical for therapeutic advancements.

Galectins play significant roles in kidney-related processes and diseases, primarily through their regulatory effects on immune responses and inflammation [10]. Among them, galectin-3 has been extensively studied for its involvement in kidney diseases. Galectin-3 facilitates profibrotic signaling and immune activation, contributing to conditions like chronic kidney disease (CKD) and acute kidney injury (AKI). It drives fibrosis by activating macrophages and fibroblasts, resulting in excessive extracellular matrix production. Elevated levels of Gal-3 have been linked to kidney fibrosis, diabetic nephropathy, and tubular injury. Experimental studies blocking Gal-3 have demonstrated protective effects, including reduced inflammation and fibrosis, suggesting its potential as a biomarker and therapeutic target in renal disorders [11,12]. Galectin-9 exhibits a dual role in kidney diseases, balancing pro-fibrotic and anti-inflammatory effects depending on the context [13]. It has been identified in kidney tissue and associated with disease modulation [14]. In nephrotoxic serum nephritis models, Gal-9 treatment reduced urinary protein excretion and crescent cell formation, likely through the induction of apoptosis in activated T-cells. Furthermore, Gal-9’s interaction with Tim-3 has been shown to reduce inflammatory damage in nephritis, emphasizing its potential therapeutic role [15,16]. Galectin-1 is implicated in the pro-fibrotic processes observed in diabetic nephropathy and CKD through the PI3K/Akt signaling pathway [17,18]. Under hyperglycemic conditions, Gal-1 is overexpressed in podocytes, leading to podocin loss and contributing to the progression of diabetic nephropathy [19]. Additionally, elevated serum Gal-1 levels are associated with hypertension, diabetes, and renal function decline, highlighting its relevance as a biomarker and therapeutic target for mitigating kidney fibrosis [20]. Although research on Galectin-4 and Galectin-7 in renal pathology is limited, both are known to modulate immune responses and epithelial homeostasis. Gal-4’s role in epithelial cells of the gastrointestinal tract suggests a potential influence on similar environments in the kidney, emphasizing the need for further investigation [21,22]. These galectins’ broader roles in inflammation and cellular adhesion underscore their potential relevance in kidney diseases.

This study aims to assess plasma levels of galectins 1, 3, 4, 7, and 9 in MM patients, exploring correlations with clinical outcomes and disease severity.

## 2. Results

### 2.1. Clinical and Demographic Characteristics

All clinical and demographic data are presented in Supplementary Table S1. Among the 47 patients included, there was a slight male predominance, with a median age of 60 years. The time between the onset of symptoms and diagnosis varied widely, ranging from 1 to 36 months. The most frequently reported symptom was bone pain, followed by general weakness and pathological fractures. According to the Durie-Salmon (DS) staging criteria, most patients were classified as stage IIIA or IIIB. In the International Staging System (ISS), out of 39 patients with available β2-microglobulin data, the majority were categorized as ISS stage 3 (51%), followed by ISS stage 1 (26%) and ISS stage 2 (23%). Serum immunofixation was performed for 38 patients, revealing the IgG Kappa subtype as the most prevalent, followed by isolated light chain subtypes (kappa or lambda).

### 2.2. Evaluation of Plasma Levels of Galectins and Biochemical Parameters

**Figure 1:**
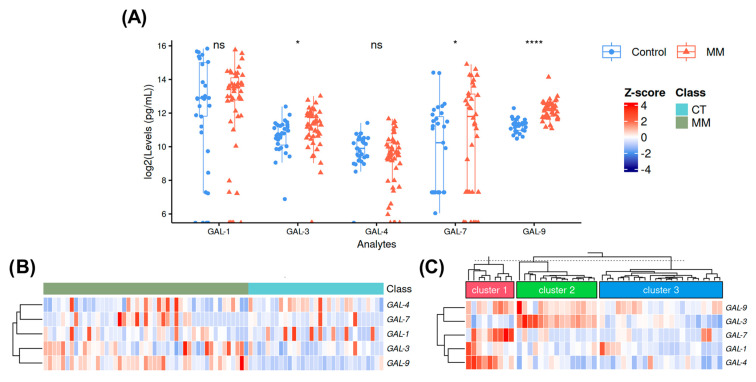
Evaluation of the plasmatic levels of galectins by boxplot (**A**) and heatmap (**B**) and (**C**) analyses. The (**B**) heatmap englobes the MM and healthy control patients. While the (**C**) heatmap represent the clusterization of MM according to their z-score values distribution. *p*-values of *t*-test are represented as follows: ns (not significant), *p* > 0.05; *p* ≤ 0.05 (*); and *p* ≤ 0.0001 (****).

Raw plasma levels of galectins and summary statistics for each are available in Supplementary Tables S2 and S3. Overall, galectin levels were elevated in MM patients compared to healthy controls. The interaction and statistical significance of galectin levels are illustrated in Figure 1. Boxplot analysis (Figure 1A) showed that GAL-1 levels were lower in MM patients compared to controls, whereas GAL-3, GAL-4, GAL-7, and GAL-9 were elevated. Among these, only GAL-3, GAL-7, and GAL-9 reached statistical significance (Figure 1A). Heatmap analysis (Figure 1B) revealed similar trends. Clustering analysis (Figure 1C) identified three distinct clusters within the MM cohort: Cluster 1 (high levels of GAL-4, GAL-7, and GAL-9; *n* = 9), Cluster 2 (elevated levels of GAL-3 and GAL-9; *n* = 15), and Cluster 3 (low to moderate levels of all measured galectins; *n* = 23).

### 2.3. Correlation Between Galectins and Clinicopathological Parameters

**Figure 2:**
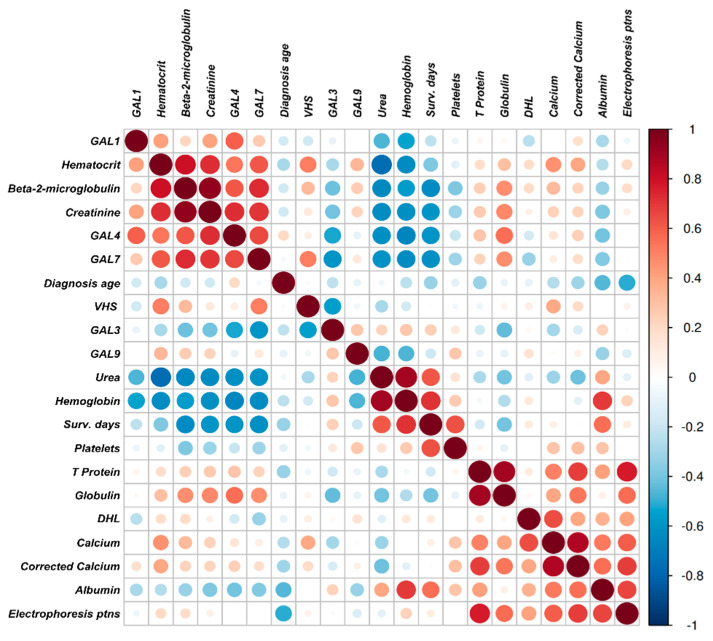
Pearson’s correlation plot between galectins and clinical variables.

The correlation analysis between serum galectin levels and biochemical parameters revealed a moderate to strong positive correlation cluster, showing statistical significance, involving Gal-1, Gal-4, Gal-7, hematocrit, creatinine, and β2-microglobulin (Figure 2; Supplementary Tables S4 and S5). A separate negative correlation cluster was observed, including urea, hemoglobin, survival days, and Gal-3. In contrast, Gal-9 did not exhibit any notable correlations. Scatter plots displaying individual correlations between galectins and key clinical variables associated with MM progression are presented in Supplementary Figure S1.

### 2.4. Cluster Analysis

When comparing clinical variables within each cluster generated by the heatmap analysis, significant differences were observed (Table 1). Notably, patients in Cluster 1 exhibited a higher incidence of clinical traits such as renal dysfunction, hypercalcemia, anemia, and fever. Hemoglobin and hematocrit levels were significantly lower in Cluster 1 (~7.6 g/dL and 23%, respectively) compared to Clusters 2 and 3 (~11 g/dL and 33–34%, respectively). Conversely, urea, creatinine, and β2-microglobulin levels were three to five times higher in Cluster 1 (105 mg/dL, 5.44 mg/dL, and 13,170 ng/mL, respectively) compared to Clusters 2 and 3 (~43 mg/dL, 1 mg/dL, and 4500 ng/mL, respectively).

### 2.5. Disease Classification and Survival

According to the Durie-Salmon (DS) classification, Clusters 2 and 3 had a higher proportion of patients in stage IIIA (87% and 74%, respectively), while Cluster 1 had a predominance of patients in stage IIIB (56%). Although there were no statistically significant differences in death rates or treatment response among the clusters, it is important to note that Cluster 1 had a 100% mortality rate, followed by Cluster 2 with 71% and Cluster 3 with 48%.

### 2.6. Gal Expression, Disease Detection, Progression, and Accuracy

**Figure 3:**
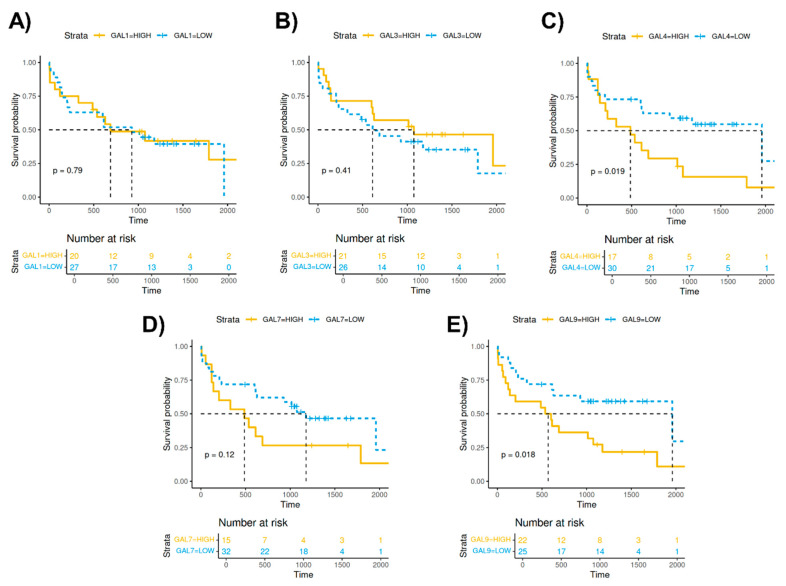
Overall survival analysis considering the high and low levels of galectins 1 (**A**), 3 (**B**), 4 (**C**), 7 (**D**), and 9 (**E**) and the survival days from MM patients.

We conducted an overall survival analysis to assess the impact of high and low galectin levels in MM patients. The analysis revealed significant associations for GAL-4 and GAL-9, indicating that higher levels of these galectins correlate with a reduced survival probability (Figure 3).

### 2.7. Galectin Levels as Biomarkers

**Figure 4:**
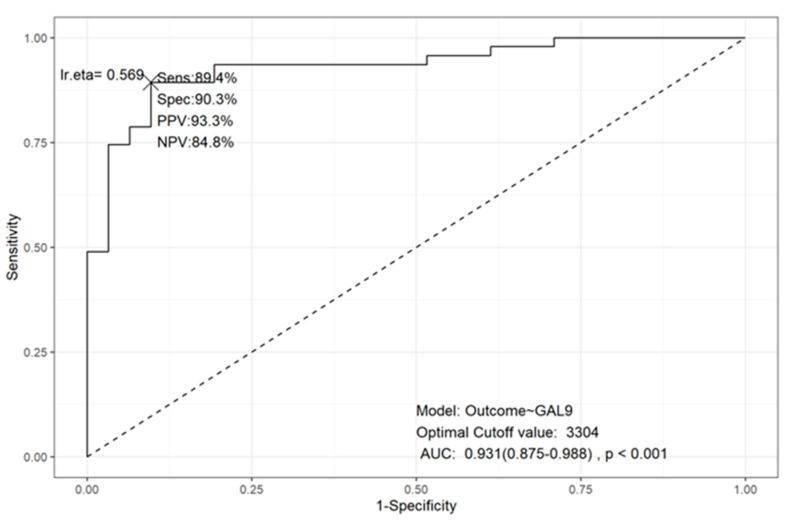
ROC plot for GLM of GAL-9 to differentiate MM and healthy patients, considering sensibility (Sens.), specificity (Spec), positive predictive value (PPV), negative predictive value (NPV), the optional fitted value (lr.eta, represented by the X letter), area under curve (AUC) and *p*-value from Wilcoxon Rank Sum test. Outcome: 1 = “MM”, 0 = “Healthy”.

To validate serum galectin levels as potential biomarkers for distinguishing MM patients from healthy individuals, we applied a Generalized Linear Model (GLM). The area under the curve (AUC) analysis demonstrated statistically significant values for sensitivity and specificity. Among the galectins tested, only Gal-9 achieved strong performance metrics, including favorable AUC and accuracy values (Figure 4), while the others showed less promising results (Supplementary Figure S2). The cutoff value for Gal-9 to distinguish between MM patients and healthy individuals was 3304 pg/mL, with a sensitivity of 89.4%, specificity of 90.3%, positive predictive value (PPV) of 93.3%, negative predictive value (NPV) of 84.8%, and an AUC of 0.931 (95% CI: 0.875–0.988), *p* < 0.001. These findings are particularly noteworthy, as Gal-9 showed both the highest plasma levels in patients and the strongest association with reduced survival probability (Figure 4).

## 3. Discussion

This study evaluated plasma galectin levels in newly diagnosed MM patients. Significant elevations of galectins 3, 7, and 9 were observed, while elevated levels of galectins 1, 4, and 7 were associated with a worse prognosis. Galectin-9 demonstrated a high capacity to differentiate MM from controls. Furthermore, elevated galectin levels correlated with renal impairment and increased disease aggressiveness, highlighting their potential as biomarkers. Galectin-3 (Gal-3) is the most studied galectin in cancer research, is involved in fibrosis and inflammation and plays a role in heart failure, renal disease, obesity and cancer [23]. In MM, Gal-3 expression in vitro has been associated with increased aggressiveness, invasion, and resistance [24]. One study found that inhibiting Gal-3 induces apoptosis in MM cells via activation of caspases 3 and 8 and poly (ADP-ribose) polymerase (PARP) cleavage [25].

To date, there are no studies specifically addressing the role of Gal-7 in the context of MM. However, research suggests that Gal-7 mediates cell-cell adhesion under physiological conditions [26]. In lymphoma, ectopic expression of Gal-7 has been associated with the metastatic potential of transplanted lymphoma cell lines, and similar correlations between tumor progression and Gal-7 accumulation have been observed in human lymphoid diseases, with no expression detected in normal tissues [27].

In our study, we observed significantly elevated levels of Gal-9 in the serum of MM patients compared to healthy controls. There was a positive correlation between Gal-9 levels and biochemical markers such as β2 microglobulin and urea, while a negative association was observed with hemoglobin. The expression of Gal-9 on neoplastic cells varies across different malignancies. In hematologic cancers, studies have demonstrated Gal-9’s involvement in the pathophysiology of various leukemias, with elevated levels correlating with more severe disease, which aligns with our findings [28,29].

Regarding MM specifically, studies have demonstrated the potential of protease-resistant recombinant Galectin-9 (hGal9) to inhibit the proliferation of several myeloma cell lines in vitro, including those resistant to bortezomib. hGal9 also induced apoptosis in patient-derived MM cells, even in those with adverse risk factors such as chromosomal deletion of 13q or translocation t(4;14)(p16;q32). In vivo, hGal9 inhibited the growth of MM cells xenografted into mice [30]. Additionally, An et al. reported significantly higher levels of Gal-9 in MM patients compared to healthy individuals, suggesting a role for osteoclast-secreted Gal-9 in negatively regulating Th1 cells through its interaction with TIM-3 on T cells [31].

The interaction between TIM-3 on T cells and its ligand Gal-9 negatively regulates the immune response. In MM patients, elevated levels of TIM-3 on CD4+ T cells, increased Gal-9 mRNA in peripheral blood mononuclear cells (PBMCs), and higher Gal-9 protein levels in serum, particularly in patients with poor prognostic indicators, have been observed. These findings are further supported by the negative correlation between serum IFN-γ and TIM-3+ Th1 cells, Gal-9 mRNA, and protein levels. Blocking the TIM-3/Gal-9 pathway in cell culture experiments restored antitumor immune function and enhanced IFN-γ secretion. These data suggest that the TIM-3/Gal-9 axis contributes to MM progression by inhibiting Th1 cell cytotoxicity and promoting Th2/Th17 cell involvement in the immune evasion of MM [32]. Therefore, this pathway could represent a potential immunotherapeutic target for MM patients.

In contrast, our analysis did not reveal statistically significant differences in serum levels of Gal-1 and Gal-4 between MM patients and healthy donors. However, both galectins, along with Gal-7, formed a cluster that correlated with disease aggressiveness parameters. This observation is consistent with Andersen et al. (2017) [33], who found no significant elevation in Gal-1 levels in MM patients at diagnosis compared to healthy controls (*n* = 102). Their study also showed no association between Gal-1 levels and bone marrow angiogenesis, clinical-pathological parameters, overall survival, or treatment response. Interestingly, the study suggested that Gal-1 plays a role in bone disease in MM, as low Gal-1 expression in vitro enhances bone resorption, and the absence of Gal-1 in the bone microenvironment accelerates the development of bone disease, indicating a regulatory role of Gal-1 in the bone marrow niche [34].

Elevated serum levels of Gal-4 have been significantly associated with various cancers, including colon, hepatocellular, and breast cancer, particularly in patients with metastasis [35]. While the specific relationship between Gal-4 and Multiple Myeloma (MM) has not yet been established, it is known that Gal-4 interacts with vascular endothelium, contributing to increased circulation of several cytokines and chemokines, such as Monocyte Chemoattractant Protein-1 (MCP-1), Granulocyte Colony-Stimulating Factor (G-CSF), and Interleukin-6 (IL-6) [36].

The interaction between transformed cells and various components of the tumor microenvironment plays a crucial role in the development and progression of MM. These interactions directly influence the clinical behavior of the disease, impacting overall prognosis [37]. In our study, we categorized patients into distinct clusters based on galectin expression. Notably, we observed that high serum levels of Gal-4 and Gal-7, along with moderate levels of Gal-1 (Cluster 3), were associated with renal dysfunction, as evidenced by elevated urea, creatinine, and beta-2 microglobulin levels. Additionally, these patients exhibited lower hematocrit and hemoglobin levels, reflecting anemia, which affects approximately 73% of those with MM [38].

Renal involvement is common in MM and other plasma cell dyscrasias. At diagnosis, around 50% of patients may present with renal issues, which are associated with increased mortality [39]. Data from the United States Renal Data System and the European Renal Association-European Dialysis and Transplant Association Registry indicate that MM patients significantly contribute to cases requiring renal replacement therapy. Notably, the overall mortality rate for end-stage renal disease in patients with MM is 58%, compared to 31% in the general population [40,41]. Galectins have emerged as important mediators in the pathophysiology of renal damage, playing critical roles in inflammatory processes, fibrosis, and tissue regeneration.

Among the most studied galectins in this context, Gal-3 has been highlighted for its relationship with the progression of chronic and acute kidney diseases. This protein regulates macrophage activation, inflammatory cytokine production, and extracellular matrix deposition, promoting renal fibrosis in conditions such as diabetic nephropathy, glomerulonephritis, and tubulointerstitial nephropathies. Gal-3 has also been linked to kidney disease in both preclinical models and clinical studies that inhibition can reduce inflammation and fibrosis, indicating its potential as a therapeutic target in progressive renal injury [42,43]. Gal-3 plays a key role in the pathogenesis of diabetic nephropathy through the a/ction of its ligand and the formation of advanced glycation end products. Blockade of Gal-3 using a natural glycoside was able to significantly inhibit macrophage infiltration, collagen accumulation and angiogenesis in the kidneys of diabetic rats [44].

In addition, other galectins, such as Gal-1 and Gal-9, have also been associated with renal context, especially in conditions of acute and chronic kidney injury. Gal-1 has been implicated in modulating the immune microenvironment, promoting tolerance and reducing inflammation. High levels of circulating Gal-1 appear to be associated with contrast-induced nephropathy and are associated with the regulation of pro-inflammatory cytokines, vascular permeability and renal injury [20], while Gal-9 is associated with regulating cell death and immune suppression in renal settings [45]. A study investigated the association between plasma Gal-9 levels, proteinuria, tubulointerstitial lesions, and renal function in patients with different stages of chronic kidney disease (CKD). The group with high Gal-9 tended to be older and to have decreased renal function, higher proteinuria, and greater interstitial fibrosis. After multivariable adjustment, high plasma Gal-9 levels were independently associated with higher stage of CKD. An analysis of gene expression in the tubulointerstitial compartment in biopsy samples showed a significant positive correlation between intrarenal Gal-9 mRNA expression and plasma Gal-9 levels [46].

Here, we identified that patients with elevated levels of Gal-1, Gal-4, and Gal-7 at diagnosis experienced worse prognosis, showing a strong positive correlation with β2 microglobulin and a negative correlation with hematocrit and hemoglobin levels. In the context of MM, β2 microglobulin is released into the bloodstream by tumor cells; its elevation indicates a higher tumor burden and increased disease activity. Elevated β2 microglobulin levels are associated with greater neoplastic cell proliferation, renal dysfunction, and poorer treatment response. This correlation was evidenced in our cohort by simultaneous increases in urea and creatinine levels. Furthermore, Gal-9 exhibited high accuracy in distinguishing between patients with MM and healthy individuals.

Our study is pioneering in demonstrating the potential of combined galectin analysis in MM, highlighting their utility as prognostic biomarkers. The levels of galectins reflected disease aggressiveness and renal impairment in MM patients. Consequently, our findings not only illustrate the association of galectin levels with disease severity in MM but also have the potential to assist in risk stratification and therapeutic decision-making.

## 4. Materials and Methods

### 4.1. Patients

Samples were collected from 47 patients diagnosed with multiple myeloma (MM) between May 2016 and January 2019 at the Hematology Service of Hospital de Câncer de Pernambuco (Recife, Brazil). All patients were included after being informed about the project and providing signed informed consent. Inclusion criteria were: age over 18 years; monoclonal gammopathy under investigation for MM and/or clinical alterations suggestive of MM; clinical fitness for a bone marrow biopsy at the posterior iliac crest; treatment-naive status, except for those using corticosteroids at doses up to 1 mg/kg/day of prednisone or up to 10 mg/day of dexamethasone for less than 10 days. The control group consisted of 31 healthy individuals matched for sex and age, with no history of immunomodulator use or previous cancer diagnosis, all of whom provided signed informed consent.

### 4.2. Laboratory Tests and Data Collection

Clinical data were collected at the time of diagnosis and throughout the study period. Biological samples were collected from patients only at the time of diagnosis. Response degrees were categorized based on International Myeloma Working Group (IMWG) criteria. Clinical and treatment data were obtained from medical records.

### 4.3. Enzyme-Linked Immunosorbent Assay (ELISA)

Plasma concentrations of galectins 1, 4, 3, 7, and 9 were determined using the sandwich ELISA technique with R&D Systems kits, following the manufacturer’s instructions. Absorbance readings were obtained using the Human Reader HS plate reader. Minimum detection limits for Gal-1, Gal-3, Gal-4, Gal-7, and Gal-9 were 150, 31.25, 62.5, 78.12, and 46.87 pg/mL, respectively.

### 4.4. Data Analyses

All data analyses were performed in RStudio IDE (2024.04.1+ 748), using different packages. The description of each analysis is displayed below.

### 4.5. Statistical Inference

Results for descriptive statistics for categorical variables associated with galectins were made using gtsummary package [47] Pearson’s correlation analysis between continuous clinical variables and Galectins were obtained using rquery.cormat function from corrplot package [48], resulting in two matrix of r and *p* values, and a correlation plot. Pearson’s correlation coefficient determined correlations between variables, categorized as weak (r = 0.3 to <0.5), moderate (r ≥ 0.5 to 0.8), and strong (r ≥ 0.8). Significance was considered when *p* < 0.05.

### 4.6. Galectins Levels

To compare galectin levels between MM patients and healthy volunteers, we performed heatmap and boxplot analyses using the ComplexHeatmap [49,50] and ggpubr [51] packages, respectively. For the heatmap analysis, the analyte level data was converted to a matrix and normalized using the scale function, which transformed the values into z-scores. For clustering analysis, we used the HeatmapAnnotation function and applied k-means clustering with three clusters across the columns. In the boxplot analysis, the values were normalized using the log2 function, and statistical significance between the two groups was assessed using a Student’s *t*-test.

### 4.7. Overall Survival

To examine whether galectin levels differed between deceased and surviving patients, we conducted an overall survival analysis using the survminer [52] and survival [53,54] packages. Patients were stratified based on galectin levels, with values above the mean classified as “HIGH” and those below the mean as “LOW”.

### 4.8. Predictive Models to Distinguish Healthy and MM Patients

We investigated whether galectin levels could differentiate between MM patients and healthy controls by performing a General Linear Model (GLM) using the glm function [55]. Each galectin was analyzed individually, along with the outcome variable (1 for MM, 0 for Control). Seventy percent of the data was used for training the model, and the remaining 30% was reserved for testing its accuracy. Model performance was evaluated using the multipleROC package [56], which provided sensitivity (Sens.), specificity (Spec.), positive predictive value (PPV), negative predictive value (NPV), optimal fitted value (lr.eta), area under the curve (AUC), and *p*-value from the Wilcoxon Rank Sum test.

## Figures and Tables

**Table 1:** Information grouped by the clusters formed by heatmap analysis. All continuous and categorical variables were evaluated by Kruskal test and Chisq test, respectivel.

Clinical Traits Based on LGAL Clusters	Overall *n* = 47 ^1^	Cluster 1 *n* = 9 ^1^	Cluster 2 *n* = 15 ^1^	Cluster 3 *n* = 23 ^1^	*p*-Value ^2^
Death	30/47 (64%)	9/9 (100%)	10/15 (67%)	11/23 (48%)	**0.021**
Survival days	811 (640)	582 (556)	823 (718)	894 (623)	0.5
Changes in renal function	14/47 (30%)	7/9 (78%)	2/15 (13%)	5/23 (22%)	**0.002**
Hypercalcemia	8/45 (18%)	4/9 (44%)	2/14 (14%)	2/22 (9.1%)	0.060
Missing values	2	0	1	1	
Anemia	25/46 (54%)	9/9 (100%)	4/14 (29%)	12/23 (52%)	**0.003**
Missing values	1	0	1	0	
Repetitive Infection	11/45 (24%)	5/9 (56%)	3/14 (21%)	3/22 (14%)	**0.046**
Missing values	2	0	1	1	
Hemoglobin (g/dL)	10.59 (3.09)	7.63 (1.32)	11.24 (2.26)	11.38 (3.36)	**0.002**
Missing values	2	0	2	0	
Hematocrit %	32 (9)	23 (4)	34 (8)	33 (9)	**0.004**
Missing values	4	1	2	1	
Urea (mg/dL)	54 (39)	105 (59)	45 (24)	41 (19)	**<0.001**
Missing values	3	1	1	1	
Creatinine	1.92 (3.50)	5.44 (6.97)	1.08 (0.71)	1.01 (0.53)	**0.002**
Missing values	2	0	1	1	
Albumin	3.50 (0.70)	3.03 (0.52)	3.73 (0.59)	3.56 (0.75)	**0.035**
Missing values	4	0	1	3	
Beta-2 microglobulin	5781 (4953)	13,170 (7529)	4915 (2909)	4085 (2847)	**0.006**
Missing values	8	3	1	4	
Durie salmon					**0.011**
IA	2/47 (4.3%)	0/9 (0%)	0/15 (0%)	2/23 (8.7%)	
IIA	3/47 (6.4%)	2/9 (22%)	0/15 (0%)	1/23 (4.3%)	
IIIA	32/47 (68%)	2/9 (22%)	13/15 (87%)	17/23 (74%)	
IIIB	10/47 (21%)	5/9 (56%)	2/15 (13%)	3/23 (13%)	
Treatment response					0.2
Death/loss of sequence	30/47 (64%)	9/9 (100%)	10/14 (71%)	11/23 (48%)	
Required retreatment	2/47 (4.2%)	0/9 (0%)	1/14 (7.1%)	1/23 (4.3%)	
RP or stable disease	5/47 (10%)	0/9 (0%)	1/14 (7.1%)	4/23 (17%)	
Still alive with disease in RC, VGPR	10/47 (21%)	0/9 (0%)	2/14 (14%)	8/23 (34%)	

^1^ n/N (%); Mean (SD). ^2^ Pearson’s Chi-squared test; Kruskal-Wallis rank sum test.

## Data Availability

The raw data supporting the conclusions of this article will be made available by the authors on request.

## Supplementary Materials

The following supporting information can be downloaded at: https://www.mdpi.com/article/10.3390/ijms252413499/s1.
