# Supplementary material for: Galectin Plasmatic Levels Reveal a Cluster Associated with Disease Aggressiveness and Kidney Damage in Multiple Myeloma Patients
Source: Int J Mol Sci. 2024 Dec 17;25(24):13499. doi: 10.3390/ijms252413499 (PMC11676459; doi:10.3390/ijms252413499)

Table S1. Clinical and Demographic Characteristics

Characteristic	N = 47[1]
**Sex**	
Female	20 / 47 (43%)
Male	27 / 47 (57%)
**Race**	
Black	10 / 47 (21%)
Non-white	31 / 47 (66%)
White	6 / 47 (13%)
**Diagnosis age**	60 (39 / 85)
**Death**	30 / 47 (64%)
**Days**	811 (4 / 2,145)
**Time (months) of the beginner of the symptoms until the first medical approach**	10 (1 / 36)
(Missing)	2
**Changes in renal function**	14 / 47 (30%)
**Hypercalcemia**	8 / 45 (18%)
(Missing)	2
**Anemia**	25 / 46 (54%)
(Missing)	1
**Thrombocytopenia**	1 / 46 (2.2%)
(Missing)	1
**Leukopenia**	2 / 46 (4.3%)
(Missing)	1
**Bone pain**	
Yes	46 / 46 (100%)
(Missing)	1
**Repetitive Infection**	11 / 45 (24%)
(Missing)	2
**Plasmocytoma**	35 / 47 (74%)
**Pathologic fracture**	25 / 46 (54%)
(Missing)	1
**Weakness**	38 / 46 (83%)
(Missing)	1
**Fever**	5 / 45 (11%)
(Missing)	2
**Weight loss**	18 / 45 (40%)
(Missing)	2
**Hemoglobin (g/dL)**	11 (6 / 20)
(Missing)	2
**Hematócrito %**	32 (17 / 60)

Table S1. Clinical and Demographic Characteristics

Characteristic	N = 47[1]
(Missing)	4
**Platelets**	259,759 (33,900 / 660,000)
(Missing)	3
**Urea (mg/dL)**	54 (18 / 222)
(Missing)	3
**Creatinine**	2 (0 / 22)
(Missing)	2
**Calcium**	10 (5 / 14)
(Missing)	4
**Albumin**	4 (2 / 5)
(Missing)	4
**Corrected calcium**	10 (8 / 15)
(Missing)	5
**T protein**	8 (3 / 15)
(Missing)	8
**Globulin**	5 (1 / 11)
(Missing)	11
**VHS (mm)**	36 (2 / 73)
(Missing)	13
**DHL**	191 (84 / 335)
(Missing)	11
**Electrophoresis Ptns**	9 (5 / 15)
(Missing)	7
**Peak presence**	37 / 43 (86%)
(Missing)	4
**Beta-2 microglobulin**	5,781 (1,438 / 20,000)
(Missing)	8
**Serum immunofixation**	
Negative	5 / 38 (13%)
Positive	33 / 38 (87%)
(Missing)	9
**Immunofixation result**	
IgA KAPPA	7 / 33 (21%)
IgA LAMBDA	3 / 33 (9.1%)
IgG KAPPA	14 / 33 (42%)
IgG LAMBDA	8 / 33 (24%)
IgM KAPPA	1 / 33 (3.0%)
(Missing)	14

Table S1. Clinical and Demographic Characteristics

Characteristic	N = 47[1]
**Myelogram (%)**	19 (0 / 96)
**Bone marrow biopsy: Presence of clonal plasmacytosis undefined**	
Inadequate result	4 / 47 (8.5%)
Negative	7 / 47 (15%)
Positive	36 / 47 (77%)
**Durie salmon**	
IA	2 / 47 (4.3%)
IIA	3 / 47 (6.4%)
IIIA	32 / 47 (68%)
IIIB	10 / 47 (21%)
**ISS**	
I	12 / 47 (26%)
II	11 / 47 (23%)
III	24 / 47 (51%)
**Treatment response**	
Death/loss of sequence	30 / 47 (64%)
Required retreatment	2 / 47 (4.3%)
RP or stable disease	6 / 47 (13%)
Still alive with disease in RC, VGPR	9 / 47 (19%)

[1]n / N (%); Mean (Min / Max)

Table S2. Raw levels of galectins in MM patients and healthy volunteers. Measurements below of the limit of the detection were considered as 0.

ID	GAL-1	GAL-3	GAL-4	GAL-7	GAL-9
MM01	21450	3966	164.25	156.25	5049
MM02	7750	4026	62.5	156.25	6449
MM03	2050	3596	756.75	156.25	4939
MM04	13850	3581	599.25	2166	4844
MM05	13950	4956	899.25	6786	6769
MM06	6750	1886	719.25	3846	2304
MM07	55550	3691	2909.25	1086	6219
MM08	0	686	0	15966	3794
MM09	4450	2496	651.75	156.25	5449
MM10	11150	4256	186.75	3306	5519
MM11	48050	2926	419.25	156.25	4474
MM12	9950	2501	246.75	2526	3889
MM13	21350	2561	696.75	5106	6424
MM14	1050	3051	681.75	156.25	2869
MM15	11250	1406	621.75	156.25	3414

MM17	7250	2851	636.75	2706	3469
MM19	8150	3696	741.75	606	4739
MM20	8050	1636	831.75	30606	7024
MM21	0	1591	0	14286	4719
MM22	12950	2291	1889.25	5886	5164
MM23	9950	1176	839.25	7206	5279
MM24	12350	701	1379.25	24906	5469
MM25	150	2781	809.25	156.25	3874
MM26	19550	2226	2129.25	19626	7314
MM27	12050	2746	1184.25	4926	5734
MM30	3550	521	666.75	1566	5894
MM31	20950	1557.333	2354.25	8526	5904
MM32	18950	1724	1364.25	19566	8209
MM33	6950	1117.333	2699.25	12486	3304
MM34	156.25	2004	576.75	156.25	3424
MM35	38650	0	3231.75	19266	2479
MM36	3650	992	1281.75	156.25	3634
MM37	13750	8252	981.75	9126	5854
MM38	8050	4252	711.75	8166	5494
MM39	22750	1472	81.75	0	3309
MM40	13050	352	779.25	0	3509
MM43	27050	912	509.25	0	3359
MM44	22850	6992	254.25	0	2144
MM45	13550	4692	194.25	0	6079
MM46	2850	2512	989.25	0	4284
MM47	12450	1952	96.75	0	6304
MM48	12250	3532	1206.75	0	4819
MM49	0	6232	1109.25	0	4129
MM50	7450	2132	1829.25	0	3114
MM51	250	4392	1011.75	0	5112
MM52	0	5072	0	0	18152
MM53	0	0	0	0	5772
CT1	14650	3831.333	1769.25	4686	2649
CT2	5450	1618	696.75	5226	3214
CT4	7650	531.3333	696.75	156.25	2594
CT9	3750	931.3333	576.75	2166	1979
CT14	30650	3031.333	959.25	2646	2794
CT26	150	1771.333	494.25	156.25	1929
CT27	7550	1978	366.75	66	2034
CT28	7350	118	1761.75	3846	2629
CT32	50450	784.6667	951.75	5466	2154
CT35	7350	2531.333	1739.25	3246	1429
CT41	58450	2131.333	1439.25	156.25	3444
CT45	5550	2811.333	1184.25	2406	2454
CT48	3550	1044.667	1469.25	966	1834
CT49	156.25	684.6667	1379.25	156.25	2764

CT50	52050	971.3333	591.75	6006	2419
CT51	1850	924.6667	1146.75	2886	2624
CT57	38950	1618	2729.25	21726	2294
CT58	0	2204.667	741.75	2946	1544
CT60	7750	2544.667	1461.75	156.25	2659
CT62	33650	2538	659.25	2346	1939
CT47	350	1151.333	1199.25	156.25	2724
CT67	850	1711.333	509.25	156.25	1734
CT70	45350	2704.667	1521.75	1206	2574
CT72	0	2464.667	1401.75	4086	3164
CT75	12650	5364.667	749.25	21366	2739
CT76	0	2311.333	501.75	156.25	2424
CT77	0	1791.333	689.25	156.25	3129
CT78	0	2411.333	1259.25	156.25	1634
CT79	42350	1364.667	854.25	156.25	2269
CT80	2350	1444.667	554.25	156.25	3514
CT81	8250	1491.333	0	156.25	5019

Table S3. Summary of galectins levels from MM patients (a) and healthy volunteers (b).

**a)**	**GAL1**	**GAL3**	**GAL4**	**GAL7**	**GAL9**
**Min.**	0	0	0	0	2144
**1st Qu.**	3600	1515	336.8	0	3572
**Median**	9950	2501	719.2	606	4939
**Mean**	12089	2722	893.3	4932	5095
**3rd Qu.**	13900	3694	1146.8	6996	5874
**Max.**	55550	8252	3231.8	30606	18152

**b)**	**GAL1**	**GAL3**	**GAL4**	**GAL7**	**GAL9**
**Min.**	0	118	0	66	1429
**1st Qu.**	600	1098	625.5	156.2	2006
**Median**	7350	1771	951.8	1206	2574
**Mean**	14487	1897	1034	3074.8	2526
**3rd Qu.**	22650	2498	1420.5	3546	2752
**Max.**	58450	5365	2729.2	21726	5019

Table S4. Correlation matrix with r values from galectins and clinical variables

	GAL1	*Hematocrit*	*Beta-2-microglobulin*	*Creatinine*	*GAL4*	*GAL7*	*Diagnosis age*	*VHS*	*GAL3*	*GAL9*	*Urea*	*Hemoglobin*	*Surv. days*	*Platelets*	*T Protein*	*Globulin*	*DHL*	*Calcium*	*Corrected Calcium*	*Albumin*	*Electrophoresis ptns*
GAL1	1																				
Hematocrit	0.41	1																			
Beta-2-microglobulin	0.21	0.8	1																		
Creatinine	0.4	0.72	0.93	1																	
GAL4	0.59	0.53	0.61	0.72	1																
GAL7	0.26	0.61	0.73	0.7	0.65	1															
Diagnosis age	-0.17	-0.29	-0.18	-0.18	0.2	-0.042	1														
VHS	-0.18	0.51	0.32	0.12	0.092	0.51	-0.026	1													
GAL3	-0.083	-0.29	-0.42	-0.41	-0.53	-0.6	-0.23	-0.55	1												
GAL9	0.0034	0.33	0.25	0.22	-0.097	0.13	-0.096	-0.058	0.27	1											
Urea	-0.47	-0.77	-0.65	-0.64	-0.62	-0.61	-0.051	-0.29	0.22	-0.48	1										
Hemoglobin	-0.54	-0.63	-0.56	-0.62	-0.66	-0.63	-0.24	-0.17	0.27	-0.47	0.89	1									
Surv. days	-0.23	-0.39	-0.63	-0.6	-0.6	-0.62	-0.33	-0.011	0.24	-0.19	0.61	0.71	1								
Platelets	-0.075	-0.11	-0.39	-0.32	-0.21	-0.31	-0.11	-0.051	0.13	0.27	0.16	0.26	0.63	1							
T Protein	0.053	0.18	0.24	0.26	0.28	0.22	-0.33	-0.064	-0.18	-0.086	-0.28	-0.049	-0.17	0.051	1						
Globulin	0.031	0.3	0.46	0.48	0.55	0.46	-0.085	0.067	-0.44	-0.1	-0.41	-0.27	-0.41	-0.1	0.89	1					
DHL	-0.25	0.18	0.19	0.08	-0.17	-0.33	-0.097	0.098	0.038	0.16	-0.12	0.13	0.11	-0.0038	0.11	0.044	1				
Calcium	0.0051	0.45	0.32	0.23	0.14	0.096	-0.26	0.38	-0.3	0.029	-0.32	0.004	0.087	0.28	0.5	0.39	0.64	1			
Corrected Calcium	0.17	0.39	0.22	0.22	0.24	0.18	-0.31	0.19	-0.15	0.08	-0.42	-0.11	0.011	0.3	0.68	0.53	0.39	0.86	1		
Albumin	-0.29	-0.26	-0.33	-0.39	-0.41	-0.38	-0.46	0.0024	0.23	-0.33	0.39	0.69	0.55	0.28	0.41	0.081	0.35	0.52	0.55	1	
Electrophoresis ptns	-0.064	0.2	0.18	0.082	0.0031	0.0093	-0.51	0.073	-0.026	-0.14	-0.11	0.23	0.1	-0.0068	0.77	0.56	0.4	0.6	0.68	0.66	1

Table S5. Correlation matrix with p values from galectins and clinical variables

	GAL1	*Hematocrit*	*Beta-2-microglobulin*	*Creatinine*	*GAL4*	*GAL7*	*Diagnosis age*	*VHS*	*GAL3*	*GAL9*	*Urea*	*Hemoglobin*	*Surv. days*	*Platelets*	*T Protein*	*Globulin*	*DHL*	*Calcium*	*Corrected Calcium*	*Albumin*	*Electrophoresis ptns*
GAL1	0																				
Hematocrit	0.068	0																			
Beta-2-microglobulin	0.35	1.5e-05	0																		
Creatinine	0.072	0.00027	6.9e-10	0																	
GAL4	0.0046	0.013	0.0032	0.00023	0																
GAL7	0.26	0.0033	0.00016	0.00038	0.0015	0															
Diagnosis age	0.46	0.19	0.44	0.44	0.4	0.86	0														
VHS	0.44	0.018	0.16	0.59	0.69	0.019	0.91	0													
GAL3	0.72	0.21	0.059	0.066	0.014	0.0044	0.31	0.01	0												
GAL9	0.99	0.14	0.28	0.33	0.68	0.56	0.68	0.8	0.24	0											
Urea	0.032	4.6e-05	0.0015	0.0018	0.003	0.0032	0.83	0.19	0.34	0.029	0										
Hemoglobin	0.011	0.0023	0.0087	0.0028	0.001	0.002	0.3	0.46	0.23	0.032	6e-08	0									
Surv. days	0.32	0.077	0.0023	0.0042	0.0038	0.0027	0.14	0.96	0.29	0.4	0.0035	0.00029	0								
Platelets	0.75	0.64	0.082	0.16	0.35	0.17	0.63	0.83	0.58	0.23	0.48	0.26	0.002	0							
T Protein	0.82	0.44	0.29	0.26	0.22	0.34	0.15	0.78	0.45	0.71	0.22	0.83	0.45	0.83	0						
Globulin	0.89	0.19	0.035	0.028	0.01	0.036	0.71	0.77	0.048	0.66	0.062	0.24	0.063	0.66	9.6e-08	0					
DHL	0.27	0.43	0.42	0.73	0.46	0.15	0.68	0.67	0.87	0.49	0.61	0.57	0.64	0.99	0.64	0.85	0				
Calcium	0.98	0.039	0.16	0.33	0.54	0.68	0.26	0.089	0.18	0.9	0.16	0.99	0.71	0.22	0.021	0.079	0.0019	0			
Corrected Calcium	0.46	0.083	0.33	0.34	0.3	0.42	0.17	0.4	0.52	0.73	0.056	0.64	0.96	0.19	0.00073	0.013	0.078	6.1e-07	0		
Albumin	0.21	0.25	0.15	0.077	0.065	0.091	0.034	0.99	0.31	0.14	0.079	0.00059	0.0092	0.22	0.068	0.73	0.12	0.016	0.01	0	
Electrophoresis ptns	0.78	0.38	0.43	0.72	0.99	0.97	0.019	0.75	0.91	0.54	0.64	0.31	0.67	0.98	4e-05	0.0079	0.071	0.0042	0.00069	0.001	0

Figure S1. Individual scatter plots from correlations between galectins 1 (**A**), 3 (**B**), 4 (**C**), 7 (**D**) and 9 (**E**), and clinical variables.

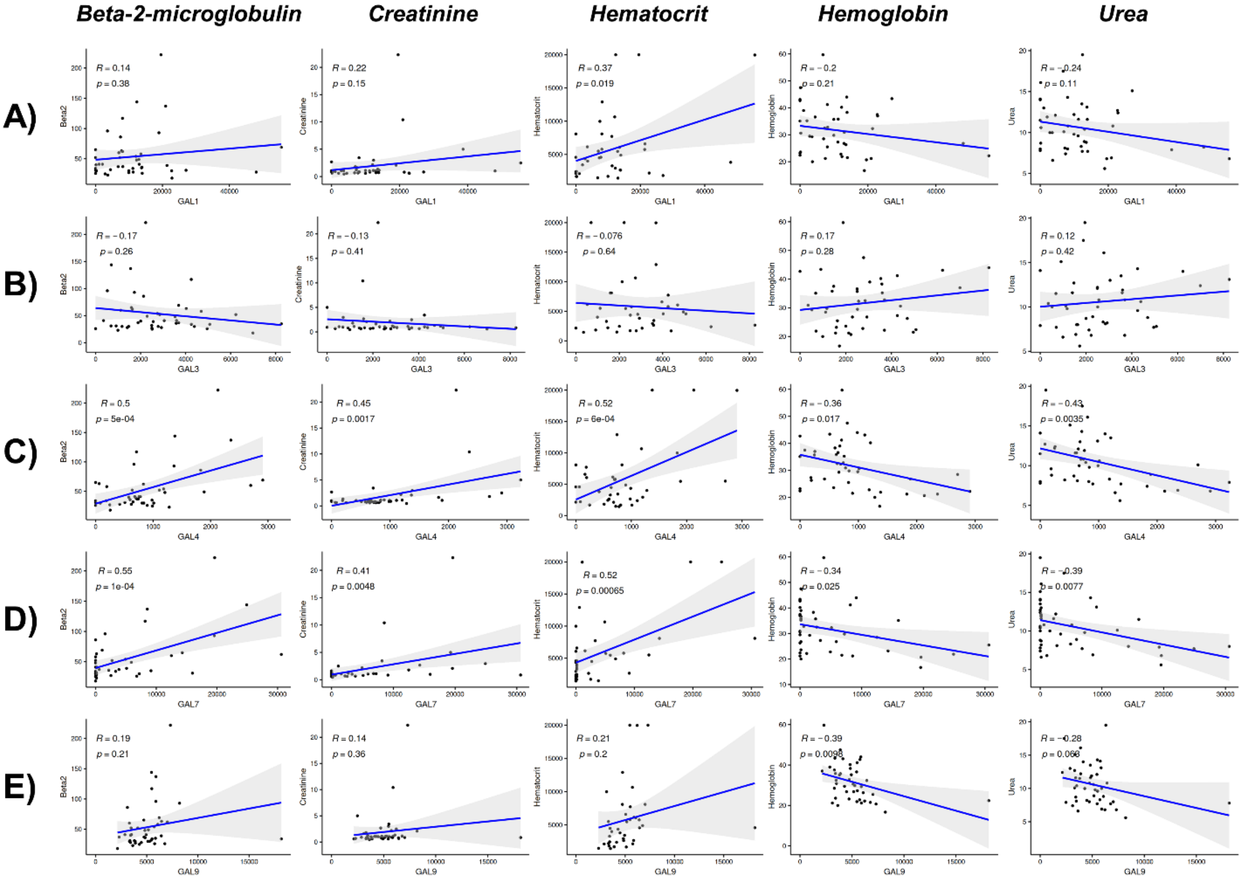

Figure S2. ROC plot for GLM of GAL-1 (A), 3 (B), 4 (C) and 7 (D) to differentiate MM and healthy patients, considering sensibility (Sens.), specificity (Spec), positive predictive value (PPV), negative predictive value (NPV), the optional fitted value (lr.eta), area under curve (AUC) and p-value from Wilcoxon Rank Sum test. Outcome: 1 = "MM", 0 = "Healthy".

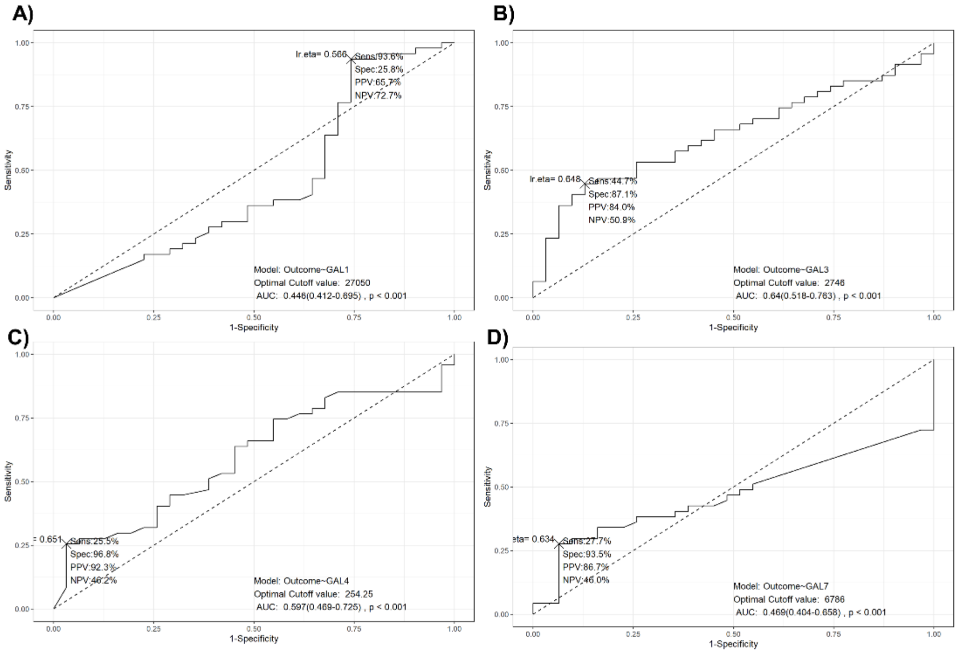

Supplement: Supplementary file 1 [file ijms-25-13499-s001.zip › ijms-3325466-supplementary.pdf]
